# Examining the determinants and efficiency of China’s agricultural exports using a stochastic frontier gravity model

**DOI:** 10.1371/journal.pone.0274187

**Published:** 2022-09-09

**Authors:** Nazir Muhammad Abdullahi, Qiangqiang Zhang, Saleh Shahriar, Muhammad Saqib Irshad, Abdullahi Bala Ado, Xuexi Huo

**Affiliations:** 1 College of Economics and Management, Northwest A&F University, Shaanxi, China; 2 School of Rural Technology and Entrepreneurship Development Rano, Kano State Polytechnic, Kano, Nigeria; 3 School of Humanities and Social Sciences, North South University, Bashundhara, Dhaka, Bangladesh; 4 Department of Economics, University of Jhnag, Jhang, Pakistan; Universidad Nacional Autonoma de Nicaragua Leon, NICARAGUA

## Abstract

This paper aims to examine the key determinants and efficiency of China’s agricultural exports with its 114 importing countries by applying the Stochastic Frontier Analysis (SFA) on an augmented gravity model for the period of 2000–2019. The Poisson Pseudo Maximum Likelihood (PPML) and the fixed effect models were also estimated simultaneously to confirm the robustness of our findings. The results reveal that China’s economic size (GDP) and its importing countries, the Belt and Road Initiative (BRI), common border, and the Chinese language positively determine China’s agricultural export flows. The results, on the other hand, also reveal that China’s agricultural export is adversely influenced by the income (per capita GDP) of China and its trade partners, currency depreciation, distance, and landlocked. On an average account, China has untapped the potential of 51% in its agriculture export with the countries used in this study. We provide policy suggestions as part of our study.

## 1. Introduction

Liberalization of international trade and globalization of the global economy has substantially aided the expansion of agricultural trade during the last decade [1, 2]. For several years, China’s economy has been rapidly expanding, with an annual growth rate of more than 10% [3]. The country’s consistent and steady economic development has made it an important worldwide economic growth engine. China, being the world’s second-largest economy, has a significant impact on global trade patterns and the global economy [4, 5]. Since its transformation into the global fastest rising economy, China has made remarkable changes in its export structure [6–8]. China’s structural changes began in 1978, when the country started its economic reform and opening-up program. China’s agriculture industry has seen a substantial transformations during the last three decades.

Chinese agricultural exports to the global market have climbed from US$2.4 billion in 2001 to US$11.27 billion in 2019, nearly two decades after China joined the World Trade Organisation (WTO). China has established itself as a major player in the world agriculture market [9]. Recent statistics show that China’s agricultural exports share accounted for a proportion of 4.33%. Although the share seems to be tiny, but China’s agricultural exports had increased considerably from 2.19% in 2001 to 3.49% in 2011 and then rose to 4.33% in 2019 [10]. Revealing that China’s exports have profited from the reduction of trade barriers such as tariffs, quantitative limitations, and other non-tariff trade measures have decreased [11, 12]. Table 1 shows that China’s agricultural export is just behind the largest exporters such as the USA, the Netherlands, and Canada, comparable with those of Germany, Brazil, Russia, and Thailand and much higher than Australia Belgium, France, and Indonesia. With profound engagement in the global market, the export performance of China did not only has a deep impact on the country’s agriculture industry, but also has a significant impact on other nations’ challenging areas. The Chinese agricultural industry contributes roughly 10% of the country’s Gross Domestic Product (GDP) and absorbs about 220 million people agricultural workers [13].

**Table 1:** Agricultural products export performance of major economies (US billion dollars).

Country	2000–2005	2006–2010	2011–2015	2016–2019
Australia	3.90	4.49	7.11	6.70
Brazil	3.22	6.41	8.99	11.12
Belgium	3.18	5.38	6.53	5.62
Canada	15.59	14.81	17.38	17.13
China	3.03	5.95	10.19	10.10
France	3.48	4.67	5.44	5.13
Germany	6.12	10.45	12.28	10.99
India	0.73	3.06	8.09	4.31
Indonesia	2.95	7.59	10.70	8.90
Japan	2.48	4.19	5.99	4.83
Malaysia	2.61	4.38	5.08	4.21
Netherlands	8.84	15.52	19.14	18.95
Russia	4.43	8.55	9.12	9.38
Thailand	3.37	7.70	11.08	10.08
USA	17.85	25.83	33.59	30.26

**Source**: Authors’ own calculation using data from UNCTAD [10].

Due to the unrealised trade potential, particularly trade in agriculture, China, like other emerging countries, will benefit from more foreign trade. Since the policy of opening up, this has piqued the interest of decision-makers. Export, according to certain research, is the most important avenue for economic integration and growth [14]. Other studies, on the other hand, have found that agricultural exports are critical to the economic success of emerging countries, including China, since the allocation of scarce resources may provide China with a competitive edge in the agricultural sector [15, 16]. As a result, a rise in agricultural revenue would directly contribute to China’s economic growth as well as the advancement of the country’s rural revitalization strategy and the war against poverty [17–19]. Xiong [20] documented that an increase in export would lead to a decrease in income inequality.

Therefore, the importance of examining the factors influencing China’s agricultural product exports become necessary, taking into account the country’s recent success in global trade as well as identifying countries where China should concentrate its efforts, countries where there is a possibility to expand agricultural export that can continue to lead the sector towards maintaining its substantial growth path. Identifying new opportunities can boost the sector’s contribution to the national economy.

Furthermore, the present study also attempts to analyze China’s agricultural sector’s exports’ efficiency. Thus, the study’s objectives are to: First, look at the factors that influence China’s bilateral agricultural export flows and how China’s BRI affects export performance. Second, estimate the efficiency of China’s agricultural exports with its importing partners. In this case, the term efficiency describes a level at which an economy or entity can no longer export additional amounts of a good, whereas, inefficient in export means an economy exports are blow the maximum level, showing a gap between the observed and maximum level of exports. Afterwards accomplishing the present study’s objectives, this study offers a significant lucrative contribution in four ways. First, our study fills a research gap in China’s agricultural export trade literature by estimating the major determinants of agriculture exports. Second, this study applies the stochastic frontier gravity model to capture China’s agriculture trade efficiencies. Third, we contribute to the growth of export research that use the Stochastic Frontier Gravity Model (SFGM), which is uncommon in the literature. Fourth, we use a panel of 114 countries to forecast China’s main trading partners over a 20-year timeframe (2000–2019).

The remainder of the paper is divided into sections: The second section of the paper presents an overview of China’s agricultural exports. We detail the methodological framework of the study in section 3. In section 4, we discuss the results of the study. Section 5 wraps up the research and makes policy suggestions.

## 2. China’s agricultural sector: An overview

**Fig 1:**
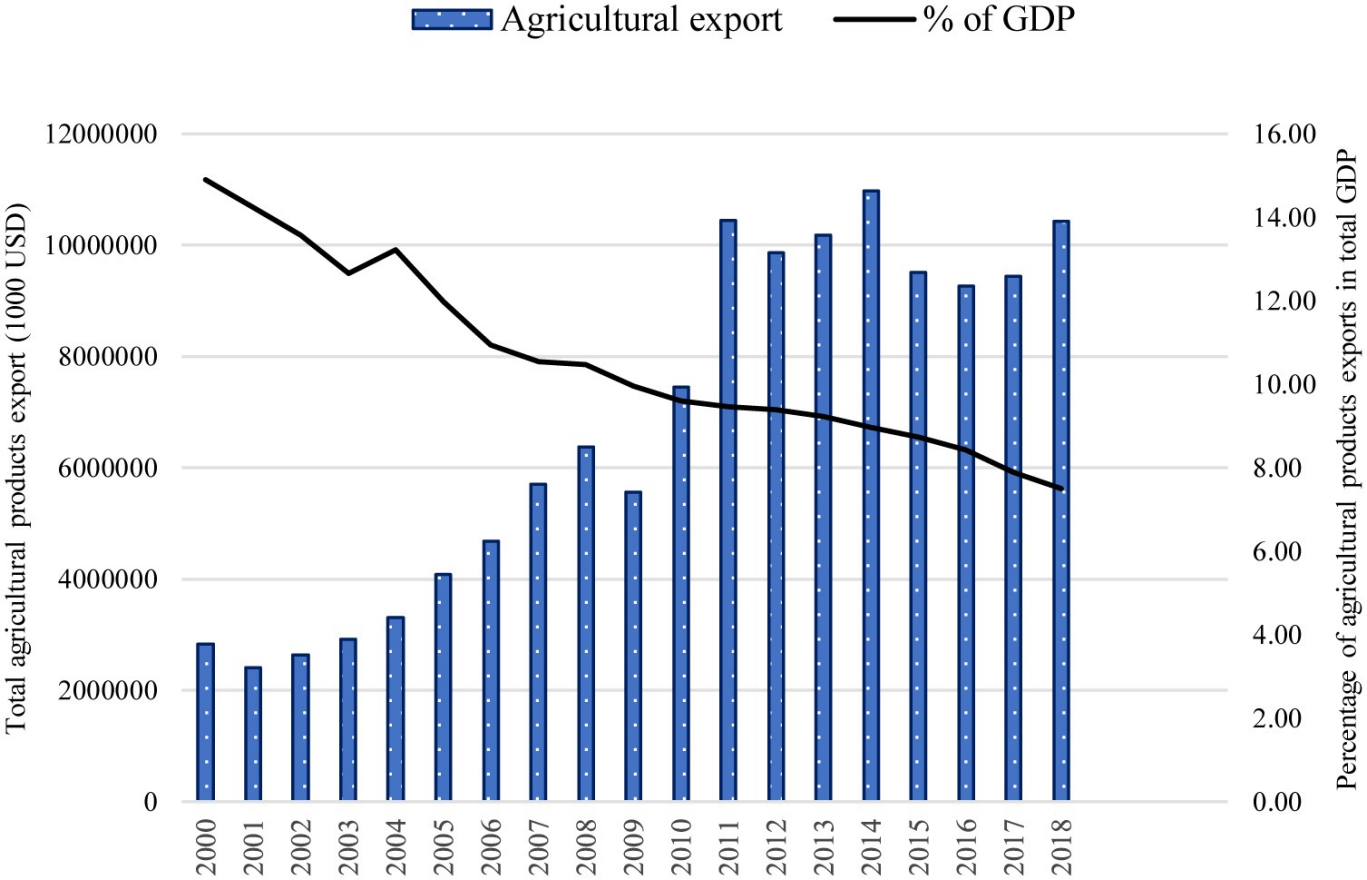
Agricultural commodities export trend 2000–2018.

A substantial majority of the population, particularly those who reside in rural areas, engages in agriculture. The agricultural sector is an important contributor to China’s economy as it contributes a considerable share of the country’s GDP and provides employment. Over the 20 years (2000–2019), the percentage share of agricultural export in total GDP was decreasing. However, on average, Fig 1 depicts the increasing trend of China’s agricultural exports during the study period. In contrast, the pattern indicates a considerable decline at times, most notably in the year after the global financial crisis of 2008. China announced a mix of macroeconomic and industrial policies to cope with the financial crisis [21]. In 2010, China’s agricultural products export rebounded with nearly 34% (Fig 1). Similarly, in 2013, Chinese President Xi Jinping introduced the Belt and Road Initiative (BRI) to the world, with the goal of encouraging deeper regional cooperation in trade and investment across Asia, Europe, and Africa, as well as their adjacent oceans [22]. Since its inception, the BRI has piqued the interest of many nations across the world, resulting in many trade prospects.

In 1998, China exported agricultural products valued at only USD 2.606 billion to the BR countries. By 2017, the exports had increased to USD 22.683 billion, nearly 10 times the export volume of 20 years earlier [23]. The BRI significantly enhances the trade network connectivity of the African countries [24].

**Fig 2:**
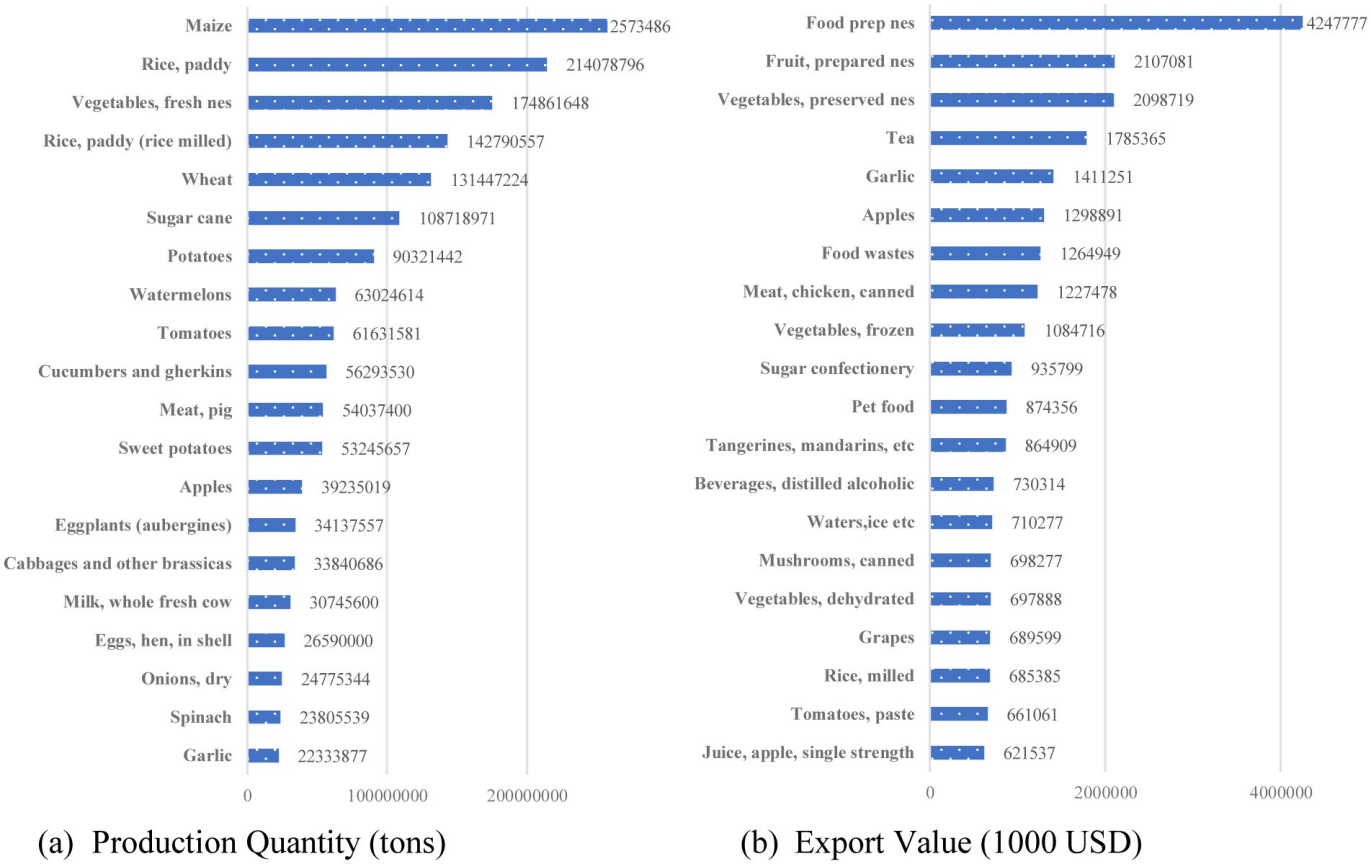
The production and exports of 20 main agricultural commodities of China in 2018. (a) Production Quality (tons), (B) Expert Value (1000 USD).

Fig 2 shows the primary agricultural produced in China and the major exportable agricultural commodities. In 2018, China produced 2.57 billion tons of maize, 2.14 billion tons of paddy rice, 1.75 billion tons of vegetables, and 1.43 billion tons of paddy (milled rice). These commodities are the top four produced in China in 2018. Similarly, the top exportable commodities are food pre, fruits prepared, vegetables, and tea with the export values of US$ 4.24 billion, US$ 2.10 billion, US$ 2.1 billion, and US$ 1.79 billion respectively.

### 2.1. The structure of China’s agricultural products market

**Fig 3:**
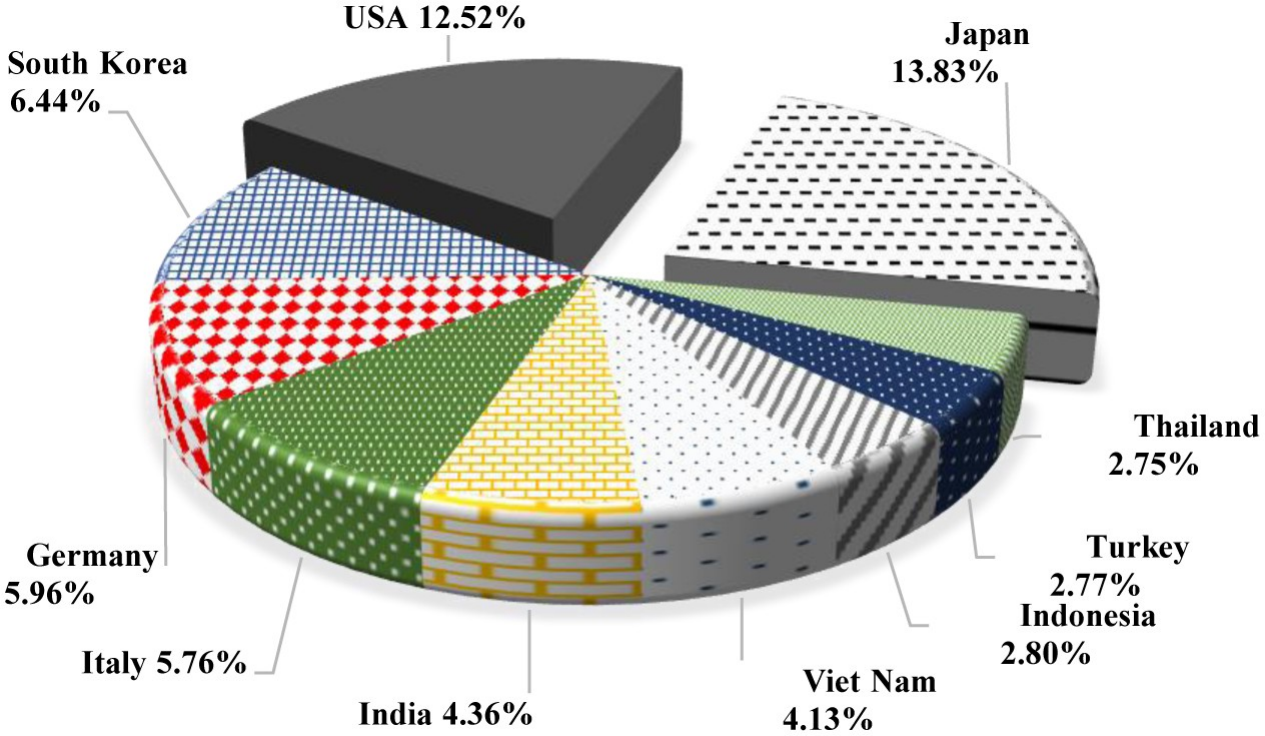
China’s top-10 largest agricultural export destination in percentage, 2000–2019.

**Fig 4:**
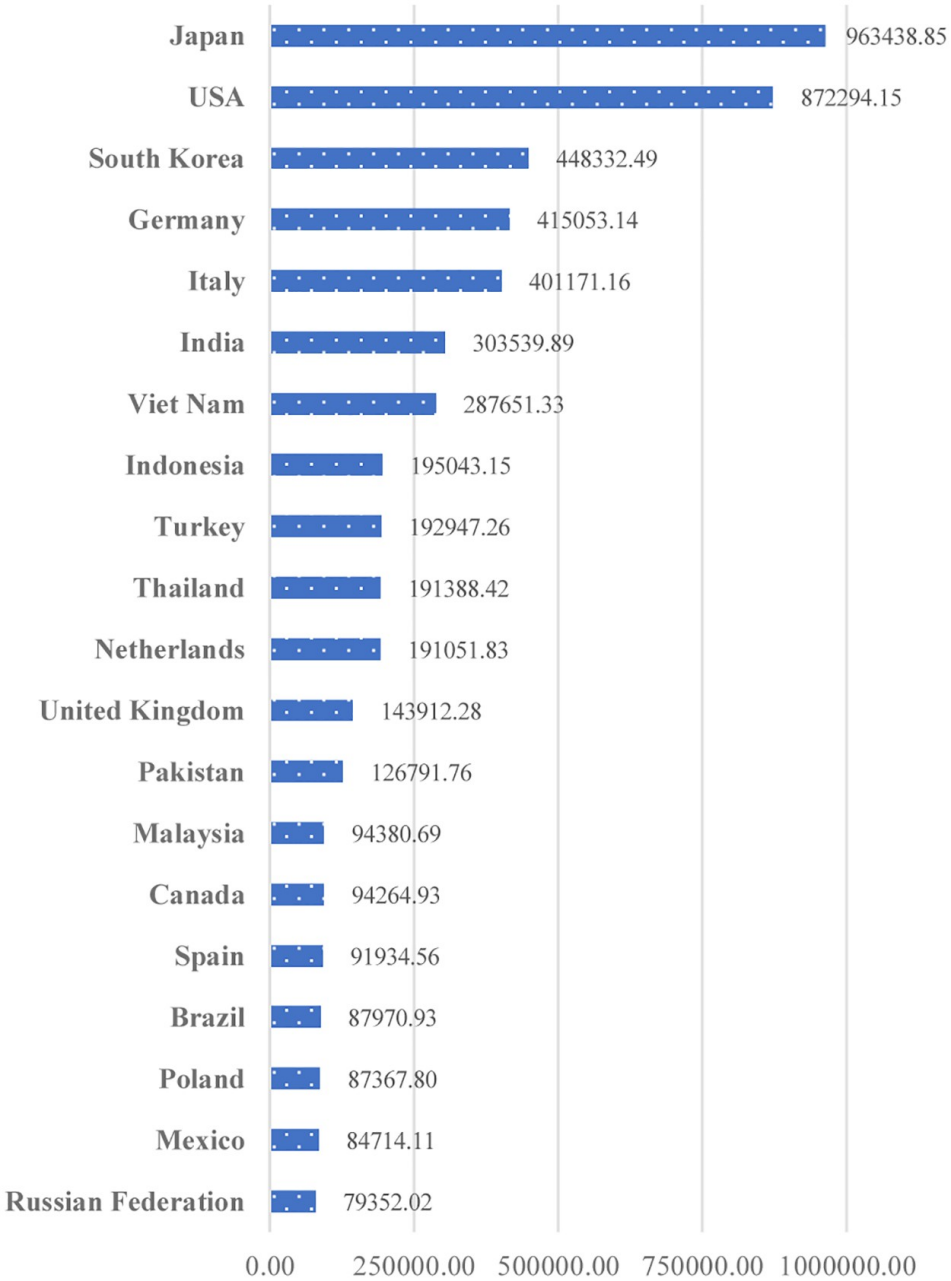
China’s average agricultural export flows in 1000 USD, 2000–2019.

Fig 3 shows China’s export destinations based on the average export share of agricultural commodity between 2000 and 2019, indicating that China’s top ten agricultural product destinations are as follows: Japan (13.83%), the USA (12.52%), South Korea (6.44%), Germany (5.96%), Italy (5.76%), India (4.36%), Viet Nam (4.13%), Indonesia (2.80%), Turkey (2.77%), and Thailand (2.75%). Similarly, China was responsible for the imports of Japan (8.33%), the USA (5.58%), South Korea (7.61%), Germany (3.11%), Italy (4.81%), India (5.97%), Viet Nam (6.61%), Indonesia (5.35%), Turkey (4.88%), and Thailand (4.43%) during the same period. Fig 3 also reveals that China’s agricultural product exports to these ten nations accounted for almost 61% of the country’s overall agricultural product exports to the international market. China’s agricultural goods have the largest foreign markets in Japan and the United States. Fig 4 reveals that these two countries together with South Korea, Germany, Italy, India, and Viet Nam imported agricultural products from China worth US$ 693 million from 2000 to 2019 annually, accounting for 53% of the total market share.

### 2.2. Literature review

This sub-section gives a detailed review of some existing literature on the determinants of exports using the gravity model. A literature review provides a complete overview of the related studies to strengthen the basis of our knowledge and to put our study in the right direction [25]. Moreover, we also divide this section into two parts. In the first part, we discuss literature on agricultural and agri-food export that uses the gravity model and or the SFGM. Meanwhile, the second part is limited to literature on commodity specific that uses the gravity model.

Recently, Ya and Pei [26] investigated the determinants of agricultural trade between China and the 58 African states using dataset from 2010 to 2019. The study explored how some factors (economic scale, geographical and demographic factors, natural resource endowment, the level of agricultural science and technology, political factors, and exchange rate factors) affect the country’s trade flows. Abdullahi, Huo [27] studied the factors and potential of Nigeria’s agri-food using the SFGM. The study shows that Nigeria’s agri-food trade potential is mostly with the big economics. Abdullahi, Aluko [28] examined the factor that influence agri-food exports from Nigeria to its importing nations (the EU), along with its efficiency and potential. The study uses a panel dataset spanning 1995 to 2019 to show that Nigerian food exports are influenced by economic size, per capita income, EU new members and distance. In addition, the study also reveals that the country’s agri-food exports to the EU are less efficient and there is a large untapped potential for trade with the EU in this sector.

Nguyen [29] observed Vietnam’s agricultural exports determinants using the SFGM on a panel dataset covering 2000 to 2018. The study revealed that the influence of “behind-the-border” constraints prevent the country from realizing its full potential. The study further revealed that ASEAN is the primary market for Viet Nam. Similarly, Hajivand, Moghaddasi [30] provided the case study of the Iranian agricultural exports. Their study indicated that almost 70% of the country’s potential has been realized during the study period.

Barma [31] investigated the Indian agricultural export with its 112 importing countries, from 2000 to 2013. The study showed that importing countries’ GDP, population, and common language increase Indian agricultural export, whereas, distance and landockedness decrease export flows. Barma proposed that the export efficiency scores can be used to develop future export diversification policies. The SFGM was used by, Atif, Haiyun [32] to investigated the key factors impacting Pakistan agricultural export. Their study confirmed the fact that the bilateral exchange rate along with tariff impact Pakistan’s agricultural export positively. Colonial history, a shared border, and a common language, on the other hand, have a detrimental impact on Pakistan’s agricultural exports. The study concluded that Pakistan has significant export potential with its neighbors and recommended that political and economic issues be resolved with nations that share a similar border.

Using a panel dataset spanning 1994 to 2008, Assem, Romstad [33] investigated the factors affecting Egypt’s agricultural exports. The study employed fixed effects and random effects to show that the GDP positively determines Egypt’s agricultural export. In contrast, GDP per capita and distance are negatively associated with agricultural export flows. Shuai [34] used a gravity model with a fixed effect method to explore the Sino-US agriculture trade potential. According to the study, China and the United States have varied agri-export potentials due to geographical differences.

Furthermore, many researchers have used the gravity model to investigate a commodity-specific model to determine the factors affecting a single commodity’s exports. For instance, Abdullahi, Shahriar [35] studied the gravity equation for Nigeria’s cocoa exports with its 36 major importing partners from 1995–2018. The study confirmed the consistence of the gravity model assumptions in the context of the Nigerian cocoa industry. The study also highlighted the needs to expand export.

Nasrullah, Chang [36] provided a case study of China’s forest product exports to the global market. Their study showed that GDP and GDP per capita positively impact Chinese export whereas distance has a negative effect on export. The study further suggested promoting the Chinese language to importing countries as a tool for increasing export flows to China’s partners.

Shahriar, Lu [6] explored the determinants of exports in the Chinese meat industry using a panel dataset spanning 1996 to 2016, for China and its importing partners. Their empirical findings showed that the GDP, Chinese language, exchange rate, and China’s land area are positively affecting the export flow of China’s pork. The study also observed the impact of the WTO and the BRI on China’s pork export flows.

Kea, Li [37] studied the dynamic form of the gravity model of Cambodian rice exports. The results revealed that the EU, ASEAN, and China are the major destinations of Cambodian rice exports. The findings also showed that the exchange rate policy, agricultural land reform, and historical ties, promote rice exports. To deal with zero trade problems, the authors applied the PPML and Heckman selection.

Using the gravity model’s static and dynamic forms, Nsabimana and Tirkaso [38] observed coffee export performance in Eastern and Southern African. The researchers found that the population size of both exporting and importing countries, geographical distances, and income are the major determinants of coffee exports. They indicated a room for expansion of coffee export by extorting countries in these regions as their finding revealed that the countries are underperforming in terms of their full potential on the international market.

In China’s context, several studies were carried out to examine the factors affecting China’s exports through trade analysis (see [4, 39, 40]). Nonetheless, just a few research on China’s agricultural exports have been discovered [26, 41–43]. To the best of our abilities, no academic study found in the existing literature that examines the factors affecting China’s agricultural product exports at a bilateral level, as well as take other time-variant and invariant metrics, to explain China’s agricultural export performance. As a result, the current study attempts to address this gap in the literature by employing a stochastic frontier gravity model.

## 3. Methodology and data

For determining the international trade flows and describe the movement of production components, the gravity model is the most commonly used. Tinbergen was the first person to proposed using the gravity equation in international trade studies [44], while Anderson [45] investigated the model thoroughly, hypothesizing that the exchange of goods and services between two nations is positively influenced by their income (economic size) and negatively influenced by their distance apart. Basically, the gravity equation is expressed as:

$$Trade_{ij}=\alpha \frac{GDP_{i}.GDP_{j}}{Dis_{ij}}$$ (1)

Where “Trade_ij_” is the value of trade between the two country *i* and *j* engaged in the transaction, GDP_i_ and GDP_j_ are the economic sizes of *i* and *j* countries. Dis_ij_ stands for the distance separates *i* and *j* countries and α is a constant. The linear version of the Eq (1) is obtained by transforming Eq (1) into logarithm form. In the case of our study, it can be stated as follows for China’s agricultural commodities exports:

$$ln\left(AGREX_{ijt}\right)=\beta_{0}+\beta_{1}\;\text{ln}\left(GDP_{it}.GDP_{jt}\right)+\;\beta_{3}\;\text{ln}\left(Dis_{ij}\right)\;+\;\varepsilon_{ijt}$$ (2)

Where “AGREX_ijt_” signifies the value of agricultural commodities exports trade from China to its importing countries. j” = 1 … 114 is for trading partners and t = 2000 … 2019 annual series. “ln” stands for a natural log.

The gravity model, which revealed the relevance of spatial dynamics in international trade theory, sparked renewed interest among academics in developing a theoretical basis for the gravity model. Anderson [45], for example, used the product differentiation model to first replicate the gravity equation. Bergstrand [46] used monopolistic competition models to investigate the theoretical foundations of bilateral trade. By assuming rising returns to scale and a differentiated product market, Helpman and Krugman [47] vindicated the gravity model. The gravity model, according to Deardorff [48] describes a wide range of models and may be validated using conventional trade theories.

Apart from theoretical reasons, the model has advanced enormously to offer more accurate and reliable estimations. For example, Rauch [49] stated that in order to explain trade patterns, language and cultural ties, as well as contiguity features, should be incorporated in the gravity model. Furthermore, Frankel, Stein [50] suggested incorporating trade agreements in the gravity model, while Cho, Sheldon [51] added the exchange rate to the gravity model. As a result, this paper extends the classic gravity model by incorporating demand and supply-side components, as well as the stimulating and resistive variables of exports. The full model specification of China’s agricultural commodities export determinants with its importing countries is specified as follows:

$${\text{lnAGREX}}_{\text{ijt}}=\beta_{0}+\beta_{1}\text{ln}({\text{GDP}}_{\text{it}}.{\text{GDP}}_{\text{jt}})+\beta_{2}\text{ln}({\text{pcGDP}}_{\text{it}}.{\text{pcGDP}}_{\text{jt}})+\beta_{3}\text{ln}({\text{Dis}}_{\text{ij}})+\beta_{4}\text{ln}({\text{Ex}}_{\text{ijt}})+\beta_{5}\text{ln}(1+{\text{Imp}}_{\text{jt}})+\gamma_{1}({\text{BRI}}_{\text{jt}})+\gamma_{2}({\text{border}}_{\text{ij}})+\gamma_{3}({\text{CN}}_{\text{ij}})+\gamma_{4}({\text{Landlocked}}_{\text{j}})+{Ϛ}_{\text{ijt}}$$ (3)

Where “*pcGDP*_*it*_ *and pcGDP*_*jt*_” are the per capita GDP of China and per GDP of its importing partners, “*Ex*_*ijt*_” is the bilateral exchange rate between China and China’s importing countries, “1 + *Imp*_*jt*_” indicates the tariff rate on China’s agricultural commodities exports. “*BRI*_*jt*_”,“*border*_*ij*_”,“*CN*_*ij*_”, and “*Landlocked*_*j*_” are dummies and stand for Belt and Road Initiative, common border, Chinese language, and landlocked respectively.

To scheme the determinants of trade for trading countries, the gravity model is often estimated. The estimate approach, however, is not without flaws. Imports and exports are frequently regarded as the sample’s average rather than the trading nations’ best potential values. It’s possible that estimating the gravity model will be difficult if the sample contains highly varied values [52]. Similarly, the model has been criticized by Anderson and Van Wincoop [53], who claims it fails to account for the influence of Multilateral Trade Resistance [MTR] on bilateral trade. Distance, tariff rate, colonial connection, and common language are all included in the MTR [54]. The standard gravity estimations, according to Ravishankar and Stack [52], are derived from the data set of nations with regular trade ties. Stochastic frontier analysis (SFA) is a para-metric approach to estimate productivity and efficiency. SFA assumes that businesses fail completely to utilize existing technology and resources, or they suffer from diseconomies of scale, leading to inevitable inefficiencies in production.

The primary drawback of the SFA is that the prospects are set using sample averages rather than the greatest potential boundaries. As a result, the stochastic frontier technique performs better in gravity model estimation. To explain trading partner variance, Kalirajan [55] added a stochastic frontier into the gravity equation. The trade frontier calculated using this method provides flexibility in determining the best amount of trade among the nations under consideration. The stochastic error term produced inside the model impacted the trade frontier positive or otherwise. This permits the randomly generated frontier to change based on the influential of the equation. The magnitude of trade observed can be compared with the projected frontier of the importing nation, in order to determine the maximum size of a trade. This method is thoroughly explained in the next sections.

### 3.1. The stochastic frontier gravity model (SFGM)

The SFA has been used extensively to evaluate firm efficiency, developed separately by Aigner, Lovell [56] and Meeusen and Van Den Broeck [57] using Stochastic Frontier Analysis (SFA) in production economics. The SFA approach argues that a given amount of inputs can yield estimates of the highest level of output and a Production Possibility Frontier (PPF). An inefficient firm/industry is one that operates on the blow frontier output, showing a gap between the observed and maximum potential levels of output. Technically efficient, on the other hand, uses the PPF to match observed and frontier output levels. As a result, the former refers to the potential for increased productivity. As a result, the technically inefficient production function describes how far the actual output does not match the potential output. The exports frontier can also be used to define the SFA approach, where inefficient export performance refers to how far actual export falls short of maximum potential export.

The benefits of using the SFGM in international trade analysis, according to Belotti, Daidone [58], are as follows: First, it gives export potential and efficiency. Second, even if a model lacks sufficient information about the missing variables, it may be used. Thirdly, it decouples the evaluation from the statistical white noise component and assesses the impact of the economic distance term, which can lead to non-normality and heteroskedasticity. Finally, it has significant theoretical implications for trade policy. The inclusion of SFA to the gravity exports equation allows the model to estimate bilateral potential exports. The error term can impact the export frontier values positively or negatively thereby allowing export frontier to vary around the influential component of the model [28, 32]. After that, the export gap may be calculated by comparing actual exports to the estimated maximum exports. As a result, the SFA results’ high theoretical and policy relevance give a compelling basis for using it. As a result, the standard gravity model using the SFA’s Eq (3) may be converted into SFGM as follows:

$${\text{lnAGREX}}_{\text{ijt}}=\beta_{0}+\beta_{1}\text{ln}({\text{GDP}}_{\text{it}}.{\text{GDP}}_{\text{jt}})+\beta_{2}\text{ln}({\text{pcGDP}}_{\text{it}}.{\text{pcGDP}}_{\text{jt}})+\beta_{3}\text{ln}({\text{Dis}}_{\text{ij}})+\beta_{4}\text{ln}({\text{Ex}}_{\text{ijt}})+\beta_{5}\text{ln}(1+{\text{Imp}}_{\text{jt}})+\gamma_{1}({\text{BRI}}_{\text{jt}})+\gamma_{2}({\text{border}}_{\text{ij}})+\gamma_{3}({\text{CN}}_{\text{ij}})+\gamma_{4}({\text{Landlocked}}_{\text{j}})+\varepsilon_{\text{ijt}}-{\text{V}}_{\text{ijt}}$$ (4)

Eqs (3) and (4) are the same, except that the error term “Ϛ_*ijt*_” is divided into two parts (*ε*_*ijt*_” and “*V*_*ijt*_). The “*ε*_*ijt*_” is the double-sided error-term and it is showing a statistical noise due to the estimation of errors with an assumption of. *N*(*0*∼*σ*^2^ *e*) The “*V*_*ijt*_” is a one-sided error-term and is truncated at 0. It is independent of “*ε*_*ijt*_” and regressors as well as a positive random variable, that measures inefficiency of exports and the value may range from 0–1. The 0 indicates that the actual exports are equal to the potential exports. Therefore, there is the absence of statistical error and there is little effect of omitting variables. If it takes a value other than 0 (that is; *V*_*ijt*_ is less than or equal to 1). This indicates that the effects of omitting variables is significant and this could impede exports. Therefore, *V*_*ijt*_ implies that export deviates from the optimal export level and this may take place due to the MTR that is usually unobservable or tough to compute and it can lead to export inefficiency performance. The parameters’ values can be empirically calculated using the maximum likelihood technique and Aigner, Lovell [56] regarded Eq (4) as pooled frontier. Not to mention that the gravity model parameters determined for the composite error term variance, $\sigma^{2}=\sigma_{v}^{2}+\sigma_{u}^{2}$ and the ratio of the inefficiency component’s standard deviation to the random error component’s standard deviation, $\lambda =\frac{\sigma_{u}^{2}}{\sigma^{2}}$, are also generated. The later determines the degree of inefficiency in comparison to random error and, if statistically significant, supports the use of the SFA method. A one-sided likelihood ratio (LR) test of the null hypothesis is used to check for the presence of technical efficiency (TE) in the model, $H_{0}:\sigma_{u}^{2}=0$, against the alternative, $H_{0}:\sigma_{u}^{2}>0$. If this hypothesis is rejected, it indicates that SFA is suitable, and if it is not rejected, it indicates that an Ordinary Least Square (OLS) model is created from the SFA model. The approach of Battese and Coelli [59] for calculating technical efficiency is used in this study. As a result, the following is the equation:

$$\text{E}[\text{Exp}\left(-{\text{V}}_{\text{ijt}}\right)|{\text{e}}_{\text{ijt}}+{\text{V}}_{\text{ijt}}]=\frac{1-\phi \left[\sigma_{*}+ϒ\left({\text{e}}_{\text{ijt}}+{\text{V}}_{\text{ijt}}\right)/\sigma_{*}\right]}{1-\phi ϒ\left({\text{e}}_{\text{ijt}}+{\text{V}}_{\text{ijt}}\right)/\sigma_{*}}\cdot \text{exp}\;[ϒ\left({\text{e}}_{\text{ijt}}+{\text{V}}_{\text{ijt}}\right)+\frac{\sigma_{*}^{2}}{2}]$$ (5)

Where ɸ (.) signifies the density function, ϒ stands for the efficiency estimated and it can range from 0–1. At this point, if the efficiency score equals to 0, then it indicates the presence of inefficiency and therefore, there is the possibility of trade with the stated factors in Eq 4. Moreover, if the efficiency score equals to 1, then there is evidence of maximum efficiency. In this case, the actual trade corresponds exactly to the potential possible trade values.

Besides calculating the ratio of the inefficiency component to the random error component $\lambda =\frac{\sigma_{u}^{2}}{\sigma^{2}}$, there are various ways to double-check the gravity model’s results. The Poisson Pseudo Maximum Likelihood (PPML) and the Fixed Effect (FE) methods are other regularly used methods to obtain gravity estimates [60, 61]. These two approaches account for unobservable heterogeneity and control the gravity model’s MTR phenomenon [62]. The FE approach, on the other hand, does not estimate the time-invariant variables of the gravity equation; as a result, variables are removed by averaging with transformation. During the estimate, factors like as distance, language, and landlocked are usually ruled out. Besides, the FE will restrict the zero trade, leading to selection bias. However, the PPML can deal with selection bias created by the zero trade problem and the presence of heteroskedasticity [63, 64]. This study also used the PPML and FE techniques for comparative analysis of our proposed model’s gravity model coefficients. The equation of our PPML is similar to Eq (3) only in PPML we do not take the log form of *AGREX*_*ijt*_. Thus, the equation can be written as:

$${\text{AGREX}}_{\text{ijt}}=\beta_{0}+\beta_{1}\text{ln}({\text{GDP}}_{\text{it}}.{\text{GDP}}_{\text{jt}})+\beta_{2}\text{ln}({\text{pcGDP}}_{\text{it}}.{\text{pcGDP}}_{\text{jt}})+\beta_{3}\text{ln}({\text{Dis}}_{\text{ij}})+\beta_{4}\text{ln}({\text{Ex}}_{\text{ijt}})+\beta_{5}\text{ln}(1+{\text{Imp}}_{\text{jt}})+\gamma_{1}({\text{BRI}}_{\text{jt}})+\gamma_{2}({\text{border}}_{\text{ij}})+\gamma_{3}({\text{CN}}_{\text{ij}})+\gamma_{4}({\text{Landlocked}}_{\text{j}})+{Ϛ}_{\text{ijt}}$$ (6)

The following is a FE equation that takes into account unobserved cross-sectional heterogeneity:

$$lnAGREX_{ijt}=\beta_{0}+\beta_{1}ln(GDP_{it}.GDP_{jt})+\;\beta_{2}ln(pcGDP_{it}.pcGDP_{jt})+\;\beta_{3}ln(Dis_{ij})+\beta_{4}ln(Ex_{ijt})+\;\;\beta_{5}ln(1+Imp_{jt})+\;\gamma_{1}(BRI_{jt})+\varepsilon_{ijt}$$ (7)

### 3.2. Sample size and data sources

The panel data set consists of bilateral agricultural products export flows from China to its 114 importing partners over 20 years, spanning from 2000–2019. During the period, the exported value of Chinese agricultural products to these 114 destinations accounted for almost 92% of China’s total agricultural products exported value. These countries were chosen based on the value of China’s agricultural exports on a yearly basis. As a result, the total number of observations in this study’s dataset is 2280 (*N* = 114 × *T* = 20). Both in terms of time and importing partners, the highest feasible frequency of data was employed. Except for dummy variables, all variables are transformed into natural log form to evaluate the relationship. Table 2 lists the data sources and descriptions of the variables utilized, whereas Table 3 gives the descriptive statistics for our model’s variables. The parameters were estimated using the Stata version 15.1 software program.

**Table 2:** Data sources of the variables used in this study.

Variables	Description	Unit	Data Sources
AGPEX_ijt_	Bilateral agricultural export	Thousand USD	UNCTAD
*GDP*_*it*_.*GDP*_*jt*_	Aggregate income	Million USD	UNCTAD
*pcGDP*_*it*_.*pcGDP*_*jt*_	Per capita income	USD	UNCTAD
Dis_ij_	Distance	Kilometers	CEPII
Ex_ijt_	Bilateral exchange rate	Yuan/J’s currency	UNCTAD
1 + Imp_jt_	1 + (import tariff/100)	Percentage	WDI
BRI_jt_	The BRI countries	0/1 Dummy	Li, Lu [4], Fan, Zhang [22]
border_ij_	The common border	0/1 Dummy	CEPII
CN_ij_	The official language	0/1 Dummy	CEPII
Landlocked_ij_	The landlocked countries	0/1 Dummy	CEPII

**Source**: Authors’ elaboration.

**Table 3:** Descriptive statistics of the variables.

Variables	Obs.	Mean	Std. Dev.	Min.	Max.
AGPEX_ijt_	2257	55854.4	156771.9	0.001	1300073
ln(AGPEX_ijt_)	2257	8.25557	2.87294	-6.90772	14.07793
ln(*GDP*_*it*_.*GDP*_*jt*_)	2264	54.31446	2.24799	47.54386	60.98749
ln(*pcGDP*_*it*_.*pcGDP*_*jt*_)	2264	16.80452	1.88317	11.69027	20.51924
ln(Dis_ij_)	2280	8.87359	0.53357	6.86276	9.86589
ln(Ex_ijt_)	2278	-0.81352	2.89251	-8.72853	4.54778
ln(1 + Imp_jt_)	2280	0.05410	0.05256	0.00000	1.00000
BRI_jt_	2280	0.25504	0.43598	0.00000	1.00000
border_ij_	2280	0.10404	0.30538	0.00000	1.00000
CN_ij_	2280	0.13082	0.33727	0.00000	1.00000
Landlocked_ij_	2280	0.01837	0.13430	0.00000	1.00000

Source: Authors’ calculation.

## 4. Empirical results and discussion

### 4.1. Factors affecting China’s agricultural products export

Table 4 displays the findings for China and its 114 importing nations based on the SFGM’s maximum likelihood estimations. The results are based on Greene [65] development of a time-invariant model (true random effects-tre). Table 5 presents the estimates using Eqs (6) and (7) (the FE, and the PPML). To confirm the appropriateness use of the SFGM, we conduct two tests. First, the statistical significance of the λ parameter supported the use of our model. As shown in Table 4, λ takes the value of 0.881 and calculates the ratio of export variations caused by inefficiency to overall export variations. It alludes to the presence of some country-specific restrictions that our proposed model does not account for. The “η” takes positive and negative signs which suggested that export inefficiency varies over time. Second, by rejecting the null hypothesis $H_{0}:\sigma_{u}^{2}=0$, against the alternative, $H_{0}:\sigma_{u}^{2}>0$, the LR test also confirms our estimation. Lastly, we further make a comparative analysis with other frequently used gravity equations (FE and PPML). However, our explanation will be mainly based on SFGM and PPML because the estimated coefficients are similar in signs, values, and levels of significance in the two models. The R^2^ value is higher in the PPML than the FE. The FE cannot estimate the impact of Dis_ij_, CN_ij_, and Landlocked_ij_ on agricultural exports of China because these variables do not change over time.

**Table 4:** Maximum likelihood estimates of the SFGM.

Variable	Coefficient	Std. Dev.	P > value
ln(*GDP*_*it*_.*GDP*_*jt*_)	1.063	0.052	0.000
ln(*pcGDP*_*it*_.*pcGDP*_*jt*_)	-0.342	0.056	0.000
ln(Dis_ij_)	-0.714	0.170	0.000
ln(Ex_ijt_)	-0.045	0.224	0.046
ln(1 + Imp_jt_)	-1.062	0.442	0.016
BRI_jt_	0.213	0.499	0.000
border_ij_	0.364	0.274	0.183
CN_ij_	1.624	0.338	0.000
Landlocked_ij_	-0.652	0.288	0.024
Constant	-36.287	2.429	0.000
η	-1.961	0.131	0.000
Λ	0.881	0.059	
LR	1797.49		
No. of observations	2244		

**Source**: Authors’ calculation.

**Table 5:** Estimates of the gravity equations.

	PPML			FE		
Variable	Coefficient	Std. Dev.	P > |Z|	Coefficient	Std. Dev.	P > |Z|
ln(*GDP*_*it*_.*GDP*_*jt*_)	0.890	0.023	0.000	2.412	0.253	0.000
ln(*pcGDP*_*it*_.*pcGDP*_*jt*_)	-0.309	0.038	0.000	-1.465	0.272	0.000
ln(Dis_ij_)	-0.907	0.045	0.000	-	-	-
ln(Ex_ijt_)	0.024	0.018	0.183	0.004	0.072	0.821
ln(1 + Imp_jt_)	-2.086	0.860	0.015	0.238	0.674	0.910
BRI_jt_	0.356	0.061	0.000	0.429	0.076	0.000
border_ij_	-0.085	0.149	0.568	0.082	1.117	0.947
CN_ij_	0.414	0.086	0.000	-	-	-
Landlocked_ij_	-1.280	0.112	0.000	-	-	-
Constant	-25.371	0.914	0.000	-98.102	9.193	0.000
Hausman test				0.002		
R^2^	0.808			0.675		
No. of observations	2244			2244		

**Source**: Authors’ calculation.

In the gravity literature GDP is used as a proxy for market size, in our case for the market size of China and its partners. China’s market size is reflecting the export capacity of agricultural commodities while the market size of the importers’ is reflecting the demand for agricultural commodities. According to the empirical estimates of the parameter (GDP), the positive and significant estimates for *GDP*_*it*_.*GDP*_*jt*_ indicate that countries with high-income levels traded more. This result is in line with the economic theory and most prior studies on gravity equations [37, 66–68]. The coefficients of this variable are 1.06 and 0.89 from SFGM and PPML respectively. This indicates that a 1% increase *GDP*_*it*_.*GDP*_*jt*_ will increase China’s agricultural exports by approximately 0.98%. China’s income and its importing partners are supporting the adequate demand and supply circumstances of agricultural commodities. We found the *pcGDP*_*it*_.*pcGDP*_*jt*_ to be highly significant at a 1% level but with negative coefficients, indicating that the negative sign of GDP per capita variable reveals that as the *pcGDP*_*it*_.*pcGDP*_*jt*_ increases, China agricultural exports less with it.

Prior international trade literature considered distance as a trade resistance factor and is anticipated to decrease China’s agricultural trade [35, 69]. The distance between Beijing and importing partners’ capital city is taken as a proxy for trade costs. In our model Dis_ij_ has a negative highly significant impact on bilateral trade. This shows that a 1% increase in distance will reduce agricultural export volume by 0.81%. This suggests that to increase its agricultural exports, China should look for a nearby market. This result is in line with the classical findings of the gravity model. Similar findings were also recorded by previous studies of agricultural product exports such as Atif, Haiyun [32]; Barma [31].

The Ex_ijt_ is used as a proxy to the exchange rate policy and it is positively significant at the 5% level in the SFGM, but insignificance in the other models. The coefficient of Ex_ijt_ shows that a 1% increment of (Yuan Chinese currency) may drop the agricultural commodities exports by 0.03%. Therefore, China needs to maintain or depreciate the exchange rate because a rise in “Yuan” is expected to drop the export revenue because of very low elasticity. China’s exchange rate policy is such a highly argumentative and extensively discussed issue that it often generates heated debate around the globe [70–72]. The United States of America has criticized China’s exchange rate policy for the two country’s bilateral trade deficit [73–75]. Evidence indicates that exchange rate volatility harms exports both in the short-and-long run [8, 76]. Thus, our result is supportive of prior studies.

The average tariff rates (1 + Imp_jt_) are an explicit measure of trade cost and higher tariff rates imposed by importing country tends to daunt export [77, 78]. In other words, the export of a country is significantly determined by the tariff rate charged by the importing country. The negative coefficients confirm that China’s agriculture exports are negatively associated with tariff rates and a 1% increase in tariff rate by importing country will decrease the agricultural exports by 1.57% and vice versa. Prior studies of agricultural exports such as Ghazalian [79] and Olper and Raimondi [80] have also reported a negative impact of this variable.

This study has also added four dummy variables to examine the effect of the BRI, border, common language, and landlocked on agricultural commodities exports. The BRI_jt_ membership of importing countries is positively significant at 1% in all models. China is building cross-border ties employing the six economic corridors under the BRI’s structure [81]. Our study found a policy variable of the BRI_jt_ to be positively significant, indicates the significance of the BRI on export flows of China agricultural commodities. The prior studies such as Shahriar, Lu [6], Kea, Li [37], Chen [82] also documented the positive impact of the BRI on China and its partners’ export flows. Under the Umbrella of the BRI China is building the global trading networks and thus regional economic incorporation through regional trade agreements, foreign direct investments, special economic zones, and infrastructural connectivity [4, 22].

The Chinese language (CN_ij_) variable is strongly significant at 1% with coefficients of 1.62 and 0.41, revealing that sharing a common language with an importing country promotes agricultural export flows by nearly 1.02%. Our study affirms other studies that recommended using the Chinese language as a tool for networks and communications, as language stimulates trade flows [83, 84]. Similar results were reported in the Chinese meat industry’s Shahriar, Lu [6] and the Chinese forest products export Nasrullah, Chang [36].

Regarding the Landlocked_ij_ variable, we found a negative coefficient of about 0.97 in the SFGM and the PPML, indicating that agricultural export flows decrease by 0.97% with the landlocked countries. As a result of being landlocked, there are additional trading expenses. Similarly, trade costs are found to have an impact on trade composition and a country’s comparative advantage in exports [85].

### 4.2. Efficiency of China’s agricultural products export

Table 6 shows the average technical efficiency of China’s agricultural export drawn from the SFGM from 2000 to 2019. It demonstrates the average export performance with major importers. The estimates show that China’s agricultural commodities export has not reached its optimum level (100% efficient) with any importing partners. Although, the majority of the importing partners have attained a relatively high efficiency. Yet, there is an excellent opportunity for improving agricultural exports’ performance with trading partners. In general, the average efficiency of the trading partners over the study period was 48.52%. While some countries are more efficient that others, for instance, the least efficient countries are Bahrain at 9.34%, Afghanistan at 16.93%, Qatar at 21.78%, and Cameroon at 26.44%. Meanwhile, higher export efficiencies are recorded for Germany 62.8%, Viet Nam 62.12%, New Zealand 61.62%, and Denmark 61.39%.

**Table 6:** Technical efficiency of China’s agriculture exports to major importing partners (%).

Partner	TE	Partner	TE	Partner	TE
Germany	62.80	Sweden	54.00	Mongolia	43.53
Viet Nam	62.12	Italy	53.98	Croatia	43.11
New Zealand	61.62	Singapore	53.97	Paraguay	42.12
Denmark	61.39	Uzbekistan	53.92	Estonia	41.74
Belgium	61.32	UK	53.90	Georgia	41.20
South Africa	61.12	S. Korea	53.56	Nepal	40.90
Spain	60.21	Romania	53.52	Cuba	40.64
Chile	59.93	Kazakhstan	53.26	Cote D’ivoire	40.38
Russia	59.91	Guatemala	53.15	Egypt	40.31
Australia	59.91	Netherlands	52.95	Turkmenistan	40.28
France	59.60	Myanmar	52.57	Slovakia	39.71
Argentina	59.55	Philippines	52.49	Ghana	39.52
Thailand	59.51	Lithuania	52.49	Senegal	39.52
Ecuador	59.42	Morocco	51.98	Oman	39.41
Portugal	59.35	Mexico	51.86	Venezuela	38.98
USA	59.31	Slovenia	51.79	Cambodia	38.85
Brazil	59.08	Nigeria	51.63	Laos PDR	38.49
Colombia	58.85	Jordan	51.29	Maldives	38.40
Sri Lanka	58.54	Kuwait	50.18	Libya	38.18
Malaysia	58.27	Pakistan	50.05	Cyprus	37.63
Austria	57.47	Canada	49.77	Togo	37.21
Latvia	56.87	Montenegro	49.65	Brunei D.	37.20
Hungary	56.64	Iran	49.62	Angola	37.03
Sudan	56.30	Dominican R.	49.58	Benin	36.59
Turkey	56.24	Belarus	49.47	Honduras	35.90
Algeria	56.23	Israel	49.26	Kenya	35.72
Finland	56.18	Ethiopia	48.96	Ireland	34.70
Ukraine	56.00	Uruguay	48.77	Tanzania	34.14
Indonesia	55.93	Bulgaria	48.25	Mozambique	30.87
Bangladesh	55.24	Costa Rica	48.25	Iraq	30.24
Japan	55.03	India	48.06	Moldova	29.72
Norway	54.87	Yemen	48.05	Lebanon	27.30
Tunisia	54.66	Serbia	47.78	Timor-Leste	27.26
Mauritius	54.56	Kyrgyzstan	46.08	Cameroon	26.44
Peru	54.51	Tajikistan	46.04	Madagascar	25.62
Greece	54.50	Czechia	45.41	Qatar	21.78
Saudi Arabia	54.25	Albania	45.05	Afghanistan	16.93
UAE	54.05	Poland	44.02	Bahrain	9.34

**Source**: Authors’ calculation

## 5. Conclusion

This study uses SFA to investigate the gravity equation of China’s agricultural exports for a panel of 114 countries between 2000 and 2019. Using the SFGM technique, we estimate the main determinants and efficiency of China’s agricultural exports with its major trading partners. However, to overcome the zero trade selection bias and the presences of heteroskedasticity and to confirm the robustness of the findings, we also employ the FE and the PPML.

The results are as follows: first, we find that China’s GDP (economic size) and its importing countries stimulate agricultural export flows. Second, we find that the BRI, common border and the Chinese language positively determine China’s agricultural export. Third, we find that China’s income (per capita) and that of China’s importing nations and exchange rates discourage agricultural exports from China. Fourth, we also observe the negative impact of geographical distance, importer’s tariff, and landlocked of importing countries. Fifth, we find that China’s agricultural exports to its 114 selected partners are largely inefficient.

We have drawn up some recommendations based on our findings. First, the research insights would improve the agricultural export flows around the globe. China introduced a number of economic and fiscal reforms. It also liberalized the trade to facilitate its global and regional integration. Therefore, China is likely to promote its agricultural exports to the global market. Second, the depreciation of China’s currency would improve agricultural export flows and its low magnitude indicates that the policymakers should give special attention to the exchange rate policy. Third, China’s agricultural exports have a satisfying effect on the BRI, therefore, China should continue to build a strong relationship with the current members and develop a policy that will attract non-members. Countries engage in agricultural trade for a variety of reasons. History shows that nations engage in agri-food trade because it yields several benefits. In the economic realm, a food exporting country may have a comparative advantage and is able to sell its food at an attractive price, thereby earning foreign currency by meeting demand in another country. Or perhaps a country wants to reduce its food surplus and support domestic prices. As a large and populous country, China enjoys comparative advantages in agricultural exports. Exports may be used to bind other nations to the exporter; in this respect agricultural exports are useful for maintaining alliances and empires. China is likely to develop its trade network under its BRI framework. To this end, the issue of the Coronavirus pandemic might hinder the growth of the Chinese agricultural exports.

Fourth, the negative effect of the geographical distance and positive effect of the Chinese language suggests that China should give more attention to the nearby countries’ markets and countries with a common language to boost agricultural commodities exports. Fifth, China’s agricultural export would benefit more from increasing its exports to countries with observed low efficiency.

Last but not the least, this study can be used as a yardstick for future researchers as it is the first piece of research that estimates the efficiency and potential of China’s agricultural exports. Further research may add novelty to the existing body of knowledge focusing on the BRI and non-BRI countries for cross-country comparisons. Future researchers may consider to include both import and export models in a single study. For comparative analysis of major agricultural exporting countries, further research efforts are needed to chain the gravity models’ findings, shown competitiveness and comparative advantage measurements of agricultural commodities in the global markets.

## Supporting information

S1 Dataset (ZIP)
